# Patients’ Perspective on Mental Health Specialist Video Consultations in Primary Care: Qualitative Preimplementation Study of Anticipated Benefits and Barriers

**DOI:** 10.2196/17330

**Published:** 2020-04-20

**Authors:** Caroline Bleyel, Mariell Hoffmann, Michel Wensing, Mechthild Hartmann, Hans-Christoph Friederich, Markus W Haun

**Affiliations:** 1 Department of General Internal Medicine and Psychosomatics Heidelberg University Heidelberg Germany; 2 Department of General Practice and Health Services Research Heidelberg University Heidelberg Germany

**Keywords:** telemedicine, remote consultation, implementation, primary health care, mental health services, thematic analysis, integrated behavioral health, health services research

## Abstract

**Background:**

Due to limited access to specialist services, most patients with common mental disorders (depression or anxiety, or both) usually receive treatment in primary care. More recently, innovative technology-based care models (eg, video consultations) have been proposed to facilitate access to specialist services. Against this background, the PROVIDE (Improving Cross-Sectoral Collaboration Between Primary and Psychosocial Care: An Implementation Study on Video Consultations) project aims to improve the provision of psychosocial care through implementing video consultations integrated into routine primary care.

**Objective:**

From the patients’ perspective, this qualitative preimplementation study explored (1) anticipated benefits from and (2) barriers to implementing mental health specialist video consultations embedded in primary care services and (3) prerequisites for interacting with therapists via video consultations.

**Methods:**

Using a purposive (ie, stratified) sampling strategy, we recruited 13 patients from primary care practices and a tertiary care hospital (psychosomatic outpatient clinic) for one-off semistructured interviews. In a computer-assisted thematic analysis, we inductively (bottom-up) derived key themes concerning the practicability of mental health specialist video consultations. To validate our results, we discussed our findings with the interviewees as part of a systematic member checking.

**Results:**

Overall, we derived 3 key themes and 10 subthemes. Participants identified specific benefits in 2 areas: the accessibility of mental health specialist care (shorter waiting times: 11/13, 85%; lower threshold for seeking specialist mental health care: 6/13, 46%; shorter travel distances: 3/13, 23%); and the environment in primary care (familiar travel modalities, premises, and employees: 5/13, 38%). The main barriers to the implementation of mental health video consultations from the patients’ perspective were the lack of face-to-face contact (13/13, 100%) and technical challenges (12/13, 92%). Notably, participants’ prerequisites for interacting with therapists (12/13, 92%) did not seem to differ much from those concerning face-to-face contacts.

**Conclusions:**

Mental health service users mostly welcomed mental health specialist video consultations in primary care. Taking a pragmatic stance, service users, who are often frustrated about uncoordinated care, particularly valued the embedment of the consultations in the familiar environment of the primary care practice. With respect to interventional studies and implementation, our findings underscore the need to minimize technical disruptions during video consultations and to ensure optimal resemblance to face-to-face settings (eg, by training therapists in consistently reacting to nonverbal cues).

**Trial Registration:**

German Clinical Trials Register DRKS00012487; https://tinyurl.com/uhg2one

## Introduction

### Primary Care Mental Health

In most countries, the prevalence of mental health conditions (eg, depression and anxiety) remains high, and the global burden of mental illness accounts for 32.4% of years lived with disability and 13.0% of disability-adjusted life-years. However, access to mental health specialists is precarious, particularly in remote and rural areas [1,2]. Patients with mental health conditions struggle with limited availability of psychosocial services and are exclusively treated in the primary care setting, which has been coined the “de facto mental health care outpatient system” [3] for the United States and other countries [3,4]. To address this care gap, clinicians, researchers, and policy makers have proposed innovative technology-based care models [6-8]. Specifically, remote consultations between patients in the primary care practice and mental health specialists by using videoconferencing have been proposed [9-11]. Given the primary care physicians’ decisive role in managing mental health conditions and the fact that most patients are treated in primary care, directly embedding mental health specialist video consultations into primary care services has been suggested [6-8,12-14]. In this regard, several comprehensive reviews indicated fairly robust evidence that interactive video consultations are an effective means to link patients in the primary care practice with specialists [6,7,15-18]. Thus, establishing a sound therapeutic alliance is generally considered to be a key driver for telepsychiatry and of upmost importance for mental health staff, including nurses [19-22]. Nevertheless, little is known about the patients’ perspective on the benefits of and barriers to mental health specialist video consultations in primary care.

### The PROVIDE Project: Fostering Cross-Sectoral Collaborations Between Primary Care Physicians and Mental Health Care Providers Through Video Consultations

This study was the first (preimplementation) phase of a larger implementation project on fostering cross-sectoral collaborations between primary care physicians and mental health care providers through video consultations (Improving Cross-Sectoral Collaboration Between Primary and Psychosocial Care: An Implementation Study on Video Consultations [PROVIDE] [23]). While we have reported results from the preimplementation phase for the primary care physicians elsewhere [24], in this paper we present findings on how to optimally tailor a video-based integrated mental health care model from the patients’ perspective. The PROVIDE service delivery model follows the integrated care approach, which virtually colocates primary care teams and specialists and has been proposed by Hilty and colleagues [7,25]. Leveraging the expertise at a distance, specialists located at their office or private practice (or a suitable, designated room at home) provide high-quality video consultations to patients presenting with depression or anxiety, or both, in the primary care practice. The intervention itself is described elsewhere in detail [26]. Recently, the PROVIDE service delivery model has been applied in a feasibility study (German Clinical Trials Register, registration no. DRKS00015812).

### Video Consultations in Mental Health From the Patients’ Perspective

There is promising evidence for the effectiveness of videoconferencing both in secondary mental health care [7,10,27-29] and in primary care [15,30]. However, with respect to crucial outcomes applied in implementation science [31], studies on the applicability of and prerequisites for rolling out mental health specialist video consultations in primary care services from the perspective of the actual stakeholders involved are rare. Indeed, it has been argued that it is necessary to involve potential users and target groups at the earliest stage possible to foster the adoption and uptake of new service delivery models [32]. Concerning the patients’ perspective, a few studies primarily evaluated patients’ satisfaction with video consultations in mental health services [33-36]. However, only 2 qualitative studies have investigated the patients’ perspective on mental health specialist video consultations embedded into primary care services [37,38]. First, Simpson [37] focused on patients’ satisfaction after a 1-year course of video consultation therapy. Second, Swinton and colleagues [38] explored the acceptability of mental health specialist video consultations prior to their implementation by investigating alliance aspects both between patients and therapists, and between therapists and primary care physicians. In sum, while studies on patients’ perspective on mental health specialist video consultations in primary care focused on the patients’ acceptance of and satisfaction with such services, they did not provide information on why patients engaged in video consultations, what kind of barriers they experienced, and whether patients had specific expectations of therapists who engage in such consultations.

### Rationale and Objectives

The purpose of this exploratory qualitative study was to take the patients’ perspective and investigate (1) anticipated benefits from and (2) barriers to implementing mental health specialist video consultations embedded in primary care services and (3) prerequisites for interacting with therapists via video consultations. Specifically, when considering anticipated barriers and benefits, we focused on both tangible (eg, journey to the primary care practice or handling technical devices) and more general aspects (eg, impact on the availability of mental health services) of the proposed mental health specialist video consultation model. With the therapeutic relationship being the main predictor for treatment outcomes in mental health care [39], we also investigated prerequisites for interaction between patients and therapists in this setting. Our findings provide in-depth information for the prospective large-scale implementation of practical and sustainable mental health specialist video consultations tailored to the needs of patients attending primary care practices.

## Methods

### Study Design and Conceptual Framework

In a naturalistic preimplementation qualitative explorative study, we conducted one-off semistructured telephone interviews to assess the patients’ perspective on implementing mental health specialist video consultations in primary care. We took a critical realist position when designing the study, analyzing the data, and interpreting the results [40]. Subsequently, we applied thematic analysis to identify shared meanings concerning anticipated benefits of and barriers to mental health specialist video consultations, along with prerequisites for interacting with therapists via video consultations [41]. All procedures performed in the study involving human participants were in accordance with the ethical standards of the institutional or national research committee and with the 1964 Declaration of Helsinki and its later amendments or comparable ethical standards. This study was approved by the Ethics Committee of the Medical Faculty at Heidelberg University (no. S-197/2017) and preregistered with the German Clinical Trials Register (registration no. DRKS00012487).

### Participants and Recruitment

We applied stratified sampling following a purposive strategy. Given the resources at our disposal, we applied sex and age as the basis for the sample stratification. Specifically, accounting for sex distributions of patient populations in mental health care, we aimed for a recruitment ratio of women to men of 2:1. To ensure appropriate representation of older people, we aimed for a 1:1 recruitment ratio of people aged 18 to 49 years to people aged 50 years or older. We consecutively recruited patients at various primary care practices in Heidelberg, Germany, and our psychosomatic outpatient clinic at Heidelberg University Hospital and asked them to participate in the study. We personally approached patients before or after their scheduled consultations. Inclusion criteria were age 18 years or older, German language proficiency, and informed consent for study participation. Exclusion criteria were poor German language proficiency, lack of informed consent, and cognitive incapability to respond to interview questions. We offered a nonadvertised individual monetary compensation of €30 (about US $35) for each interview.

### Data Collection

To capture participants’ descriptions systematically, we designed a semistructured question interview guide (Multimedia Appendix 1) that was reviewed after the first 3 interviews. Since the interview guide proved to be coherent and comprehensive, no changes were made. During March and April 2018, the interviews were conducted by the second author (MH, a PhD student focusing on video consultations in primary care, master’s degree in sociology, expertise in qualitative research), who had no contact or relationship with any participant prior to the study. We obtained written informed consent from all individual participants prior to study enrollment and guaranteed the absence of nonparticipants during the interview. All interviews were audio recorded. In the interviews, we first asked participants about their experience with the health care system, particularly about seeking help for mental health conditions. Second, we verbally described the mental health specialist video consultation model, including that the patient would be located in the primary care practice while the mental health specialist would consult from her or his office or private practice or a suitable, designated room at home (Multimedia Appendix 2). Third, we discussed benefits, barriers, and prerequisites for interacting with therapists via video consultations. Fourth, we collected sociodemographic data from the participants and supplemented the audio data with field notes produced during the interviews. In between the interviews, MH discussed the progress of sampling and data collection with the first author (CB, MD, resident in psychosomatic medicine) and the last author (MWH, MD, internal medicine specialist and master’s degree in clinical psychology), for example, with respect to the sample composition and the level of data saturation. We did not repeat any interviews.

### Data Analysis

After the audio recordings were transcribed verbatim by a professional transcription service (Transkripto, Rotterdam, Netherlands), we anonymized the data. We did not return the transcripts to the participants for comments. Two coders (CB and MH) independently conducted a computer-assisted thematic analysis in MAXQDA 12 (VERBI GmbH) of 3 individual interviews. At this stage, the coders developed the code system following an inductive (bottom-up) approach by paraphrasing, generalizing, and abstracting the original data [41]. We subsequently applied this code system to analyze the remaining 9 transcripts top-down. To ensure that all key aspects were represented, we discussed newly derived themes and modified the codes when necessary. Theme saturation was reached when the analyzed data did not provide any new themes or meaning of themes, that is, when the inductively developed themes represented and covered all the data [42]. Both coders compared their analyses and resolved disagreements in a final code system. Multimedia Appendix 3 summarizes the key themes, including definitions and supporting quotes. We followed the consolidated criteria for reporting qualitative research (COREQ) guidelines for reporting qualitative study results [43]. All materials were translated from German to English for this paper by CB and MWH.

### Member Checking

To support the credibility of our analyses and review data saturation, we conducted member checking with the participants [44]. To this end, the participants received an anonymized written summary of the interview findings and evaluated to what extent these findings reflected their statements. We contacted all participants via telephone to receive their feedback on the summary that we afterward accounted for when discussing the results.

## Results

### Sample

We approached 37 people (34 from outpatient clinics, 3 from primary care practices) with an initial interest in scheduling an individual telephone interview. We could not reach 6 patients at the indicated phone number or email address. A total of 18 patients declined to be interviewed after having received further study details, most frequently due to a lack of interest (n=9) and lack of time (n=6). In sum, we conducted individual telephone interviews (range 29-66 minutes, mean 38 minutes) with 13 participants consenting to study participation.

Table 1 [45] presents the sociodemographic characteristics of the 13 participants. Table 2 depicts the cross-tabulated table for the sample stratification by sex and age.

**Table 1:** Sample description (N=13).

Characteristic		Values
**Age (years)**		
	Mean (SD)	48.7 (17.0)
	Range	21-77
Female sex, n (%)		8 (62)
**Education (years), n (%)**		
	>9	5 (39)
	≤9	8 (62)
Married, n (%)		8 (62)
**Degree of urbanization of the place of residence^a^, n (%)**		
	Cities (densely populated areas)	2 (15)
	Towns and suburbs (intermediate-density areas)	9 (69)
	Rural areas (thinly populated areas)	2 (15)
Duration of interviews (minutes), mean (range)		39 (29-66)

^a^Stratified according to the degree of urbanization classification, 2018 version, of the European Commission [45].

**Table 2:** Cross-tabulated table for the sample stratification by sex and age.

Age range (years)	Female	Male
18-49	5	2
≥50	3	3

### Themes and Subthemes

We identified 3 key themes and 10 subthemes. Within the key themes, we stratified findings along the corresponding subthemes. In the presentation of the results below, we first describe participants’ anticipated benefits from the integration of mental health specialist video consultations into primary care services. Second, we discuss the barriers anticipated by the participants. Third, we discuss related prerequisites for interacting with providers via mental health specialist video consultations in greater detail. Finally, we review our findings considering the member checking results.

### Participants’ Anticipated Benefits

Participants identified several benefits in 2 specific areas: in the accessibility of mental health specialist care (shorter waiting times: 11/13, 85%; lower threshold for seeking specialist mental health care: 6/13, 46%; and shorter travel distances: 3/13, 23%); and in the primary care environment (familiar travel modalities, premises, and employees: 5/13, 38%). These benefits with corresponding quotes are presented in the following.

#### Shorter Waiting Times

Participants (11/13, 85%) expected more rapid access to specialist care for mental health conditions due to the coordinating role of the referring primary care physician. They also envisioned a more seamless treatment trajectory, particularly that primary care physicians and mental health specialists would work more closely with each other. Moreover, patients anticipated better treatment outcomes than they would have had with the current practice in connection with shorter waiting times:

They [the mental health specialist video consultations] will save time and offer immediate aid. If you are affected and you suffer from depressions and you don’t know what to do and they give you a list [of therapists] and you call and don’t get any answer, the depression gets worse. So, this solution [the mental health specialist video consultation] is phenomenal, in my opinion...So, you go to your primary care physician and shortly at least you have someone, who shows you the way.

Participant 11, female, in her 50s

#### Shorter Travel Distances

Some participants (3/13, 23%) expected a shorter travel distance as a further aspect of an improved access to specialist mental health care. They argued that patients could save additional time and effort, as in most cases the primary care practice would be in their hometown or nearby. Thus, long-distance travel or additional driving to a mental health specialist who maintains a practice in a larger city would not be necessary:

And yes, as already mentioned, you are in a familiar environment and you know how to get there. And in case of an emergency, you can be brought by someone, or picked up, because for us [in the countryside], this is easier than driving into the city or being brought there by someone.

Participant 08, female, in her 30s

#### Lower Threshold for Seeking Specialist Mental Health Care

Besides the abovementioned more tangible benefits of improved accessibility of mental health specialist care, some participants (6/13, 46%) described a psychological effect of the model:

I think there are many people who may benefit from this [mental health specialist video consultations]. As mentioned, this—the problem with mental health conditions—is steadily increasing. Maybe for some people, those 4 or 5 consultations are already enough...And maybe people who would not start actual therapy would make use of it...I think for those people, it would be somewhat facilitated.

Participant 06, female, in her 30s

The participants highlighted that the mental health specialist video consultations could decrease the threshold for accessing mental health care and therefore help to overcome the stigma of seeking professional psychosocial support for persons (1) with mild mental health conditions, (2) with no experiences with psychosocial care, and (3) unwilling to attend a face-to-face consultation with a mental health specialist.

#### Familiar Primary Care Environment

Several participants (5/13, 38%) appreciated that the proposed model would leverage the familiarity of the primary care environment. When engaging in mental health specialist consultations, patients would already know the practice staff, the premises of the practice, and the travel modalities to the practice:

The fact that it [the mental health specialist video consultation] takes place in the primary care practice, I think, this is a familiar environment for the patient. Therefore, one already knows the way to get there; you know what it looks like in there. For some people, it is probably an obstacle to go somewhere unfamiliar. Here [when engaging in mental health specialist video consultations] you already know the practitioner; you have already known the medical assistant for a longer time.

Participant 08, female, in her 30s

The staff in the primary care practice could encourage the patient to engage in the video consultations and assist him or her with handling the videoconferencing platform. The participants viewed the primary care physician’s involvement in the model as crucial, since the participants assigned him or her a key role in (1) managing their health problems (eg, knowing the patient and their medical history and social circumstances in detail) and (2) referring them to medical specialists.

### Anticipated Barriers

Despite their generally positive attitude toward video consultations, participants also anticipated barriers, some of which were specifically tied to the proposed model itself (lack of face-to-face contact: 13/13, 100%; technical challenges, such as an unstable internet connection: 12/13, 92%; organizational challenges: 3/13, 23%), while other barriers were related to the acceptance of psychosocial services as such the stigma of seeking mental health care (7/13, 54%).

#### Lack of Face-to-Face Contact

All participants (13/13, 100%) mentioned the lack of personal face-to-face contact with the mental health specialist as a major concern. Participants imagined “talking to a screen” as impersonal and uncomfortable, especially when discussing sensitive issues:

It [therapy] often becomes painful, doesn’t it? Therefore, depression comes with emotions. How can we deal with emotions while sitting in front of a computer?

Participant 11, female, in her 50s

Physical contact, for example, through common gestures such as the mental health specialist handing a handkerchief to the patient, would be impossible. Referring to their own personal experiences with using Skype, FaceTime, etc, some participants argued that the mental health specialist would miss several aspects of the body language, even with facial expressions and gestures being visible:

Well, I experience it myself when conducting Skype calls with familiar people. Although you speak to someone you know well, it still feels different. The distance between the person and you is different from personal encounters. In addition, the whole gesture and body perception—how you pose questions and so on—how does one express oneself? Facial expressions and other things are not conveyed during such a Skype call.

Participant 07, male, in his 50s

Although participants valued the possibility of using the mental health specialist video consultations as an initial consultation with a mental health specialist or for tackling emergencies, it was clear to some participants (5/13, 39%) that they would insist on having a regular face-to-face consultation with the specialist at some point:

Let me say, for emergencies, initial contacts, or coordinating steps clarifying what to do next, this [mental health specialist video consultation] is okay, but at some point, I must speak to the doctor in person.

Participant 11, female, in her 50s

#### Technical Challenges

Almost all participants (12/13, 92%) related several challenges to the specific technology used for conducting mental health specialist video consultations. Some participants brought up technical limitations, such as an unstable internet connection, insufficient technical features at the office, insecure data protection, or high demands on potential users who are still unfamiliar with the use of computers and videoconferencing (the so-called digital immigrants, that is, individuals born before the widespread adoption of digital technology [46]):

Concerning the technical aspect, technologies like Skype and video calls have not truly reached the elderly. In addition, for older people, it may simply be strange to talk to a screen. For younger people, I think, this is no problem at all.

Participant 08, female, in her 30s

#### Organizational Challenges

Some participants (3/13, 23%) also expressed concerns that the implementation of mental health specialist video consultations would require time and spatial resources and therefore profoundly impact on the workflow of the primary care practice. However, the descriptions of the organizational challenges were rather general:

I have my doubts, because I believe the primary care physicians are the biggest problem. They need time for this. They need additional rooms. They must deal with this whole thing. This is an additional task that doctors then must handle.

Participant 02, female, in her 60s

#### Stigma of Seeking Mental Health Care

Several participants (7/13, 54%) contemplated that, independent of the acceptance of technology, people may not participate in mental health specialist video consultations due to their negative stance on mental health care as such. Participants elaborated that personal preferences and the societal view on both mental health conditions and psychosocial support may impede the acceptance of the model:

In my opinion, this [the refusal of mental health care] is not necessarily related to video consultations. I think that this is a general stance on mental health care.

Participant 13, female, 40s

Besides benefits of and barriers to the integration of mental health specialist video consultations in primary care, participants also elaborated on prerequisites for the therapeutic relationship in the context of mental health specialist video consultations. We address this aspect in the following subsection.

### Prerequisites for Interacting With Providers in Video Consultations

Notably, participants’ prerequisites for interacting with mental health specialists in video consultations did not seem to differ much from those concerning face-to-face contacts. In fact, none of the participants related the prerequisites for the mental health specialist to the setting of the mental health specialist video consultation model, that is, speaking to the mental health specialist via videoconferencing. In contrast, all participants (13/13, 100%) emphasized aspects concerning the general therapeutic relationship independent of the therapeutic setting:

The doctor should be competent, charismatic, and sensitive. He should give me the feeling that I matter and that someone is listening to me. He should know what he is talking about and give me the feeling that I am being looked after. Trust is very important. I need mutual trust. If I do not trust the doctor, I will not see him.

Participant 07, male, in his 50s

Participants repeatedly named empathy, being taken seriously, and feeling appreciated as necessary prerequisites establishing a trustful relationship with the mental health specialist. Two interviewees wished for the possibility of choosing either a female or male mental health specialist. All in all, participants’ primary concern was to feel comfortable with the mental health specialist and to be able to establish a good relationship.

### Member Checking

Of the 13 participants, 7 (54%) agreed to take part in member checking. Of these 7 participants, 6 confirmed that their personal stance expressed in the interviews was adequately reflected in the final consolidation of the results. One participant had developed a more negative view of the mental health specialist video consultation model since the initial interview. He had started entertaining privacy concerns with engaging in video consultations in the familiar primary care environment (eg, concerns that medical assistants might breach confidentiality).

## Discussion

### Principal Findings

We investigated the patients’ perspective on mental health specialist video consultations in office-based primary care accounting for both tangible and more general factors. In sum, participants viewed the mental health specialist video consultation model positively and anticipated several fairly strictly defined benefits: (1) improved accessibility of mental health specialist care by shorter waiting times, shorter travel distances, and a lower threshold for seeking specialist mental health care; and (2) the familiar environment in the primary care office with familiar practice staff, as well as familiar travel modalities and premises. Notably, all these benefits were specifically linked to the specific feature of the mental health specialist video consultation model: the integration of mental health specialist video consultations directly into the primary care practice. However, participants also mentioned some barriers, namely the lack of face-to-face contact, technical challenges, and organizational challenges for the practice staff. Somewhat different from the professional stance that clinicians conducting video consultations require special skills, such as to be trained with the technology [47], participants’ prerequisites for the mental health specialist conducting video consultations did not differ from the prerequisites applied to face-to-face contacts, namely a trustful and appreciative therapeutic relationship. Taken together, our findings suggest that the integration of mental health specialist video consultations into primary care practices may be a promising care model that warrants further investigation in interventional studies.

### Limitations

First, at this stage, our main goal was to identify and address potential threats to the successful adoption of mental specialist consultations in primary care in a qualitative preimplementation study [48-51]. Due to the nature of the qualitative approach we applied, the generalizability of these findings may be limited. However, qualitative methods have been promoted as a particularly effective means to involve potential users of a new service in developing interventions for mental health conditions, such as when considering the implementation in routine practice [52-55]. Moreover, we aimed at enhancing the transferability by thoroughly describing the research context and methods following established standards for the reporting of qualitative studies.

Second, as is common in preimplementation studies, we relied on self-reports in order to tailor the intervention for planned feasibility and effectiveness trials. Nevertheless, self-reported intentions and practice, which the participants reported in the interviews, may, to some extent, differ from participants’ actual behavior, due to issues of social desirability, low self-awareness, comprehension, and accurate recall or prediction [56]. In this respect, it is somewhat likely that the benefits, barriers, and prerequisites mentioned in our study differ to some degree from those that participants would observe after having experienced mental health specialist video consultations. Hence, to avoid an attitudinal fallacy, we must be very cautious in inferring situated behavior from the verbal accounts described in our study [57]. In fact, in a real-life situation, people may have different or more difficulties with mental health specialist video consultations than those reported in this study. While we have tried to minimize the constraints of self-reports, such as by firmly reassuring participants of the confidential nature of their participation, given the limited validity and reliability of self-reported behavior, it is both necessary and beneficial to study actual behavior in future interventional studies. To this end, we have embarked on a feasibility study (PROVIDE-B, German Clinical Trials Register registration no. DRKS00015812), with a protocol that was informed by the results presented in this paper [26].

Third, the anticipated benefits and barriers noted by our participants may not be representative of the larger population. Even by qualitative standards, our sample size was rather moderate. While it has recently been shown that 9 interviews are sufficient for code saturation, our sample size bears some risk that we did not reach meaning saturation, in that we may have missed more subtle conceptual issues [58-60]. However, with respect to our goal to identify specific benefits and barriers, we were primarily concerned with code saturation, which ensures “a comprehensive understanding of explicit concrete issues in data” [59]. Moreover, our sampling frame was somewhat limited to patients from our outpatient clinic, which may have introduced volunteer bias. Since these patients already had at least the experience in seeking mental health care at the outpatient clinic, they might have evaluated the mental health specialist video consultation model from a more experienced viewpoint. Thus, our findings may be biased to an overall positive attitude toward the model, as it may improve the accessibility of mental health specialist care, which is the main challenge in the current system. Future studies could apply higher incentives to stratified sampled service users presenting to general practices, for which the mental health specialist video consultations are directly intended.

Fourth, the interviewer was a young (ie, a so-called digital native), technologically open-minded PhD student whose dissertation is on implementing video consultations in primary care. While we cannot fully rule out that this constellation might have biased the study findings in favor of the benefits from video consultations, we continuously tried to ensure self-reflexivity by using field notes before and member checking after the data analysis. Moreover, by adhering to the COREQ reporting standards, we aimed at maximum transparency in documenting the research process.

Fifth, we did not assess whether and, if applicable, to what extent the study participants had any experience with using video chats (eg, FaceTime or Skype). While it is known that 57% of people aged 10 years or older in the German population use the internet to make video calls [61], we cannot determine whether familiarity with video calls in general may have had an impact on the study participants’ attitudes toward mental health video consultations.

Sixth, to support the credibility of our analyses, we conducted member checking with the participants. Notably, 1 of the participants altered her view of the model and expressed a more negative attitude toward mental health specialist video consultations. This is well known as a difficulty in achieving credibility through member checking, since participants may provide different accounts of their experiences or opinions during the interview and the member checking [62]. Since all other participants whom we contacted for member checking approved our summary of their interview statements, the member checking underpinned the credibility of our analyses.

### Comparison With Prior Work

#### Benefits: Improved Accessibility and Familiar Primary Care Environment

Our findings support the growing consensus that incorporating telehealth into primary care services will likely allow patients to access their usual health care providers more easily [27]. Clearly, our work adds that—from the patients’ perspective—the 2 key arguments for embedding mental health specialist video consultations into primary care practices are the improved accessibility of mental health care services and the familiar environment of the practice. First, our participants stated that patients would not have to travel long distances to get in touch with a mental health specialist, as the primary care physician’s office is often in the local community or at least nearby. This is in line with previous findings [63-65]. Second, participants anticipated that integrating mental health specialist video consultations might also save patients from long waiting times, since immediate scheduling of consultations after the first contact with the primary care physician would be possible. This assessment is consistent with findings from a clinical trial on joint teleconsultations [66]. Trial participants emphasized the convenience of joint consultation appointments and that punctuality was better in the teleconsultations than in hospital outpatient clinics. However, the problem of the pervasive limited availability of providers seems to be still evident with the mental health specialist video consultation model, as specialists would employ time slots previously reserved for in-person contacts. Hence, waiting times could remain a challenge for both modes of delivery. Nevertheless, it seems highly plausible that, at least for some patients, early or crisis intervention through mental health specialist video consultations could reduce the need for further specialist care in the long run and eventually save resources. Finally, participants in our study argued that familiarity with the primary care practice staff and the localities of the practice provide an encouraging environment, in which the patient feels comfortable to engage in mental health care. While such an engagement still depends on the perceived stigma [67,68], the anonymity and flexibility that the presented model allows bear great potential to reduce this very stigma for patients reluctant to reach out to a specialist in person [69,70].

#### Barriers: Challenges for the Practice and Lack of Personal Contact

First, the articulated barriers (eg, unstable data connections and insufficient technical equipment in the individual practice) underline that a high technical usability and comprehensive training should be ensured prior to the implementation of video consultations in primary care practices [52]. Concerning the anticipated difficulties in certain patient groups (eg, digital immigrants), 1 study showed that age may be a significant factor in whether a patient would accept having video consultations [70]. This study examined patients’ interest in a telehealth model in which they would meet a health care provider from their home via a video call. In this regard, our model could facilitate handling unfamiliar technology, because patients would be instructed and supported by specifically trained practice staff.

Second, the participants had reservations about the organizational resources needed in the primary care practice when integrating the mental health specialist video consultations into routine care (eg, spatial requirements and additional workload for the practice staff). In this respect, some researchers have considered it essential to have in-depth familiarization with the organizational workflows of the respective primary care practice (eg, staff working patterns, room requirements, and practice management) prior to the development and implementation of video consultation services [71,72].

Third, the participants were concerned with the lack of face-to-face contact when reverting to video consultations—an observation that several previous studies of videoconferencing conducted in specialty medical settings have reported [38,70]. Obviously, most participants upheld the personal encounter as a core value, preparing the ground for a trustworthy doctor-patient relationship. Nevertheless, some patients may appreciate the physical separation introduced by the mode of delivery as described in a qualitative study exploring young people’s perspectives on receiving psychiatric services via video consultations [73]. Specifically, participants in the study suggested that the new format may alleviate patients’ anxiety before a meeting with a specialist. They regarded the format as less intimidating than being in the same room with the therapist. At any rate, a recent randomized clinical trial has shown that, despite the physical separation, patient-provider communication in telemedicine is not inferior to communication during face-to-face consultations [74].

In sum, despite the anticipated barriers, 11 of our 13 participants were willing to engage in the proposed mental health specialist video consultation model. In contrast to other studies, in our study none of the participants perceived security and privacy concerns related to video consultations as a barrier [65,73,75]. This might have resulted from our assurance that data security and end-to-end encryption were mandatory features when describing the model to the participants.

#### Prerequisites for Interacting With Providers in Video Consultations

While the participants consistently highlighted the importance of the therapeutic relationship for mental health specialist video consultation models, prerequisites for interacting with specialists in video consultations did not differ from those relevant for face-to-face consultations. Namely, patients expected that therapists should prepare the groundwork for a respectful, appreciative, and trustful therapeutic relationship. In contrast to previous findings [38], in our study, participants did not indicate that a personal encounter prior to the first video consultations would be required to establish such a relationship. One explanation for this observation could be that the participants considered the primary care physician to be a “trusted gatekeeper” who facilitates the encounter with the therapist.

### Conclusions

Our study showed that mental health service users mostly welcomed innovative technology-based care models such as mental health specialist video consultation–integrated care. Taking a pragmatic stance, service users, often frustrated with uncoordinated care, may consider such models to bear great potential to increase access to mental health care. Specifically, service users value their embedment in the familiar environment of the primary care practice. At the same, they demand minimal technical disruptions during video consultations and optimal resemblance to face-to-face settings by therapists consistently capturing and addressing nonverbal cues. While this main observation may be relevant for clinicians already conducting video consultation as part of routine care, our work contributes to preparing the ground for feasibility and effectiveness trials accounting for our preimplementation results during the development of interventions. In perspective, interventional studies will (1) shed light on the actual behavior of service users and professionals engaging in technology-based care models and (2) determine target patient groups who would maximally benefit from such models.

## Appendix

Multimedia Appendix 1 Semistructured guide for telephone interview.

Multimedia Appendix 2 Guideline for verbal presentation of the mental health specialist video consultation model.

Multimedia Appendix 3 Summary of themes and subthemes.

## Abbreviations

COREQ: consolidated criteria for reporting qualitative research
PROVIDE: Improving Cross-Sectoral Collaboration Between Primary and Psychosocial Care: An Implementation Study on Video Consultations
