# Supplementary material for: Patients’ Perspective on Mental Health Specialist Video Consultations in Primary Care: Qualitative Preimplementation Study of Anticipated Benefits and Barriers
Source: J Med Internet Res. 2020 Apr 20;22(4):e17330. doi: 10.2196/17330 (PMC7199141; doi:10.2196/17330)
Supplement: Multimedia Appendix 1 [file jmir_v22i4e17330_app1.docx]

**Multimedia appendix 1 –**

**Semi-structured guide for telephone interview**

*(Finalized version as of August 2017)*

*A. Current health care in general and for patients with mental health conditions*

- What counts for a good medical treatment from your point of view? What are the important aspects?
- In ideal case, how would such a treatment, session or consultation proceed?
- Consider situations in which you were not able to attend appointments with primary care physicians, psychiatrists or psychotherapists. What were the most common reasons?

*B.* *Introduction to the* [REDACTED FOR MASKED REVIEW] *treatment model*

- Do you have any remaining questions concerning the treatment model?
- What do you think of the proposed treatment model?
- Would you be willing to receive treatment conducted in accordance to the model mentioned above?
  - What problems, risks or difficulties can you imagine?
- Concerning feasibility, practical implementation and success of treatment…
  - How could the treatment be optimized to tackle arising problems?
  - What are potential advantages?
  - What are potential disadvantages?
- Which aspects do you personally consider as important regarding video consultations?
- To what extent do you expect yourself or other patients to benefit from treatment in accordance with the treatment intervention as mentioned above?

*C. Interview termination*

- Do you have any further questions?
- Are there any remaining aspects important to you that we have not addressed yet?
