# Supplementary material for: Patients’ Perspective on Mental Health Specialist Video Consultations in Primary Care: Qualitative Preimplementation Study of Anticipated Benefits and Barriers
Source: J Med Internet Res. 2020 Apr 20;22(4):e17330. doi: 10.2196/17330 (PMC7199141; doi:10.2196/17330)
Supplement: Multimedia Appendix 2 [file jmir_v22i4e17330_app2.docx]

**Multimedia appendix 2 –** **Guideline for the verbal presentation of**

**the mental health specialist video consultation model**

To tailor the mental health specialist video consultation model according to patients` needs prior to its actual implementation, we presented a first draft of the model to all study participants. The draft featured the following intended **core intervention components** in line with the UK Medical Research Council (MRC) guidance on the process evaluation of complex interventions:

- Localisation of the patient: the patient will be situated in the primary care practice.
- Localisation of the mental health specialist: the MHS will be located in her or his office/private practice or a suitable, designated room at home.
- Type of communication: Patients and MHS will communicate via real-time video consultations only. E-mail or telephone may only be used to schedule appointments or in case of technical failure or interrupted connectivity.
- Technical configuration: We will ensure the highest level of technical and procedural data protection by using a secure (i.e., encrypted), web-based videoconferencing platform on a subscription basis.
- Frequency of the video consultations: Patients will receive five consultations in biweekly intervals.
- In addition, we discussed the following **contextual factors** with the study participants: Specific roles and tasks of the primary care physician, the mental health specialist, and the practice staff (e.g., presence of the primary care physician during video consultation)
- Spatial aspects (e.g., requirements for the consultation room)
- Intensity of the video consultations (e.g. duration of the consultation)
