# Supplementary material for: Patients’ Perspective on Mental Health Specialist Video Consultations in Primary Care: Qualitative Preimplementation Study of Anticipated Benefits and Barriers
Source: J Med Internet Res. 2020 Apr 20;22(4):e17330. doi: 10.2196/17330 (PMC7199141; doi:10.2196/17330)
Supplement: Multimedia Appendix 3 [file jmir_v22i4e17330_app3.docx]

**Multimedia appendix 3 – Summary of themes and subthemes**

| *Key theme* | *Definition* | *Subtheme* | *Supporting quotes* |
| --- | --- | --- | --- |
| Anticipated benefits from the model | Anticipated benefits regarding feasibility, implementation and treatment success of the proposed MHSVC model | Shorter waiting times | “Yeah, I think that is a great idea and alternative. Well (..) not to wait too long for the appointment. And you just go to the family doctor.” [Participant 10] |
|  |  | Shorter travel distances | “And that you know how to get there and, if necessary, you can be driven, picked up again, because it is at least easier, as if I drive now somewhere to the city and someone must bring and pick me up again.” [Participant 08] |
|  |  | Lower threshold for seeking specialist mental health care | “My mother constantly gets this tranquillizer prescribed by her GP. This [the MHSVC] would take away this barrier for her, since she just bring herself to see a specialist […] if this were just next door and the GP would take her there… this would make sense. She would get this kind of help much faster. […] It just doesn’t take so much, rather one can check out in five sessions or so if the patient is in greater need [for support] or can be managed by the GP […]” [Participant 05] |
|  |  | Familiar environment in primary care | “As it takes place in the family doctor's practice, I think that it is a familiar environment for the patient.” [Participant 13] |
| *Key theme* | *Definition* | *Subtheme* | *Supporting quotes* |
| Anticipated barriers for the model | Anticipated barriers regarding feasibility, implementation and treatment success of the proposed MHSVC model | Lack of face-to-face contact | “Impersonality is the greatest disadvantage, yes, because in the end you speak with a picture [laughs]. You don't see the body language of the doctor either. Yes, you only see, I would say, the face.” [Participant 11] |
|  |  | Technological challenges | “But if you have someone, who has never communicated on the screen before, it won't work.” [Participant 12] |
|  |  | Organizational challenges | “They (the GPs) don’t have any space for this.” [Participant 01] |
|  |  | Stigma of seeking mental health care | “Yes, either because it's strange for you. If someone says, you should have psychotherapy and with that, some people already have a problem.” [Participant 13] |
| *Key theme* | *Definition* | *Subtheme* | *Supporting quotes* |
| Patients’ prerequisites on the patient-provider interaction | Prerequisites on the mental health specialist (e.g. characteristics, qualifications) | None | “The first impression, in other words the first two, three sentences, is crucial for the following conversation. […] This is, as I believe, a very crucial / And if you manage to do that, with the first three sentences, that the ‘chemistry’ is just right, that you bridge the gap, then it is fine.” [Participant 12]  “I would find it more sensible that it’s always the same psychotherapist, because in the end you build up a certain kind of trust and a connection with such a person.” [Participant 10] |
